# Structures of Pathological and Functional Amyloids and Prions, a Solid-State NMR Perspective

**DOI:** 10.3389/fnmol.2021.670513

**Published:** 2021-07-01

**Authors:** Asen Daskalov, Nadia El Mammeri, Alons Lends, Jayakrishna Shenoy, Gaelle Lamon, Yann Fichou, Ahmad Saad, Denis Martinez, Estelle Morvan, Melanie Berbon, Axelle Grélard, Brice Kauffmann, Mathias Ferber, Benjamin Bardiaux, Birgit Habenstein, Sven J. Saupe, Antoine Loquet

**Affiliations:** ^1^CNRS, CBMN UMR 5348, IECB, University of Bordeaux, Pessac, France; ^2^CNRS, INSERM, IECB, UMS 3033, University of Bordeaux, Pessac, France; ^3^CNRS UMR 3528, Institut Pasteur, Paris, France; ^4^CNRS, IBGC UMR 5095, University of Bordeaux, Bordeaux, France

**Keywords:** prion structure, amyloid fibril, solid-state NMR, functional amyloids, structural biology

## Abstract

Infectious proteins or prions are a remarkable class of pathogens, where pathogenicity and infectious state correspond to conformational transition of a protein fold. The conformational change translates into the formation by the protein of insoluble amyloid aggregates, associated in humans with various neurodegenerative disorders and systemic protein-deposition diseases. The prion principle, however, is not limited to pathogenicity. While pathological amyloids (and prions) emerge from protein misfolding, a class of functional amyloids has been defined, consisting of amyloid-forming domains under natural selection and with diverse biological roles. Although of great importance, prion amyloid structures remain challenging for conventional structural biology techniques. Solid-state nuclear magnetic resonance (SSNMR) has been preferentially used to investigate these insoluble, morphologically heterogeneous aggregates with poor crystallinity. SSNMR methods have yielded a wealth of knowledge regarding the fundamentals of prion biology and have helped to solve the structures of several prion and prion-like fibrils. Here, we will review pathological and functional amyloid structures and will discuss some of the obtained structural models. We will finish the review with a perspective on integrative approaches combining solid-state NMR, electron paramagnetic resonance and cryo-electron microscopy, which can complement and extend our toolkit to structurally explore various facets of prion biology.

## Introduction

**FIGURE 1:**
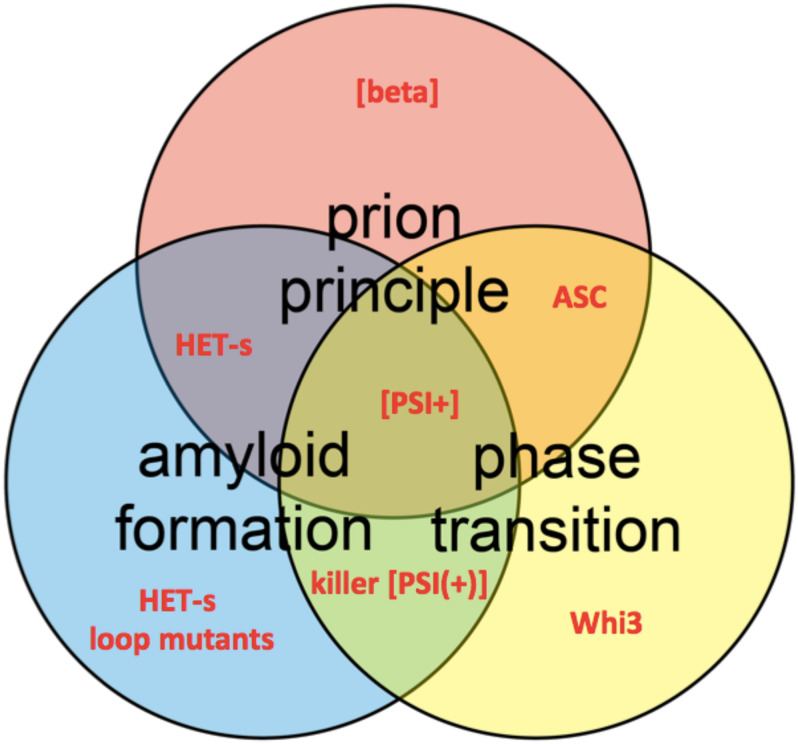
Venn diagram shows the conceptual intersections of amyloid formation, prion principal, and phase transition. Several protein and prion examples are highlighted in red, such as HET-s loop mutants (Daskalov et al., 2014), the prions [beta] (Roberts and Wickner, 2003), [PSI+] (True and Lindquist, 2000), and killer [PSI(+)] (McGlinchey et al., 2011a) and the ASC assembly (Cai et al., 2014).

Prions are infectious proteins, representing the most recently uncovered category of pathogens (Prusiner, 1982). Prion proteins can undergo a refolding (or misfolding) toward an alternative, highly cooperative polymeric conformational state, which is able to self-replicate by serving as a structural model or template for the soluble molecules of the protein (Collins et al., 2004). The term *prion* (proteinaceous infectious particle) emerges from the biomedical field as the causative agent of several neurodegenerative diseases, known as transmissible spongiform encephalopathies or TSEs (i.e., bovine Scrapie, bovine Spongiform Encephalopathy or ‘mad cow disease,’ and human Creutzfeldt-Jakob disease and Kuru) (Prusiner, 1982; Aguzzi and Calella, 2009). Most prions have been described as proteins having the ability to self-assemble into amyloid fibrils (Wickner, 2016; Scheckel and Aguzzi, 2018). Amyloids are ordered fibrillar aggregates formed by the stacking of β-sheet structural elements. However, not all prions are amyloids, as for instance recently reported for the yeast prions [GAR+] (Brown and Lindquist, 2009) and [SMAUG+] (Chakravarty et al., 2020), and the prion [beta] (see Figure 1; Roberts and Wickner, 2003). Likewise, only few amyloids have been described as entities having a prion capability (Sabate et al., 2015).

Although *bona fide* prions (prions having full infectious life cycle from host to host) are rare (Scheckel and Aguzzi, 2018), the ability of a protein to form self-perpetuating aggregates appears to be at play in a much broader range of human pathologies including Alzheimer’s disease (AD), Parkinson’s disease and amyotrophic lateral sclerosis (ALS) (Aguzzi, 2009; Prusiner, 2012; Ayers and Cashman, 2018). The prion principle (the emergence and self-propagation of a protein state) is not exclusively associated with misfolded proteins causing pathologies (see Figure 1) but underlies various physiologically important cellular processes like long-term memory consolidation and antiviral innate immunity in animals (Hou et al., 2011; Cai et al., 2014; Si and Kandel, 2016). Such protein-based phenomena are associated to the emergence of so-called *functional amyloids* (Rayman and Kandel, 2017; Loquet et al., 2018b) and have been equally proposed to play a role in an epigenetic control of flowering in plants (Chakrabortee et al., 2016).

Prions have been identified and explored in a variety of microorganisms, notably in yeast, filamentous fungi and more recently in bacteria and viruses (Wickner et al., 2015; Yuan and Hochschild, 2017; Nan et al., 2019). The term ‘prion’ has been introduced in fungal genetics for the first time in the 90s, where the prion phenomena represent a form of protein-based inheritance (Wickner, 1994). The formation of self-propagating aggregates by the yeast prion proteins generally leads to a loss-of-function of the native protein and subsequently the appearance of a novel phenotype, which can be transmitted to daughter cells and meiotic progeny by cytoplasmic inheritance (Liebman and Chernoff, 2012; Tuite, 2013). Environmentally responsive or integral part of a bet-hedging evolutionary strategy, yeast prions appear widespread in nature and are involved in diverse set of cellular processes ranging from control of gene expression and translational termination to cell morphology determination (Halfmann et al., 2012; Liebman and Chernoff, 2012; Holmes et al., 2013). Nevertheless, as the phenotypes caused by most yeast prions result from loss-of-function of a protein, which already performs a distinct molecular role in its native state, the functional character of the yeast prions has been contested and an alternative view presents them as analogous to human amyloidosis and other prion diseases (Wickner et al., 2011). Yet, another fungal prion – [Het-s] – from the filamentous ascomycete *Podospora anserina*, has been established as a functional prion controlling programmed cell death (PCD) in the context of conspecific non-self recognition (Saupe, 2011; Debets et al., 2012).

In spite of the importance for human health and the urgency to understand fundamental biological processes, the molecular mechanisms of prion emergence (nucleation) and infectivity (propagation) remain elusive, hindered by the difficulty to obtain detailed molecular structures of prion and prion-like folds. From structural point of view, protein complexes (or aggregates) behaving as prion-like entities could represent molecular assemblies of individually folded domains or strictly cooperative folds, like amyloids, where monomers contribute toward β-sheet-rich folds by layering inside the same structures, extending into fibrillar macromolecular assemblies (amyloid fibers). Nevertheless, amyloid formation is only one facet of a broader physico-chemical process termed *phase transition* (Boeynaems et al., 2018; Mathieu et al., 2020; Figure 1). Pursuing a better understanding of the prion phenomena, solid-state nuclear magnetic resonance (SSNMR) spectroscopy has been a technique of predilection to investigate crucial structural information about amyloids and prions (Tycko, 2011; Meier et al., 2017; van der Wel, 2017; Loquet et al., 2018a). Here, we will review the structural features of pathogenic and functional amyloid fibrils, which rely within their pathological or biological cycles on the prion principle, and how SSNMR data can be used to obtain structural models of such fibrils. Because our aim is not to provide an exhaustive list of all amyloid structures solved by SSNMR, we will highlight several well-documented amyloid fibrils for which SSNMR has played a key role in understanding the structural architecture.

## Misfolded Protein Aggregates Are Structural Hallmarks of Pathological Diseases

### The Cross-β Fold

**FIGURE 2:**
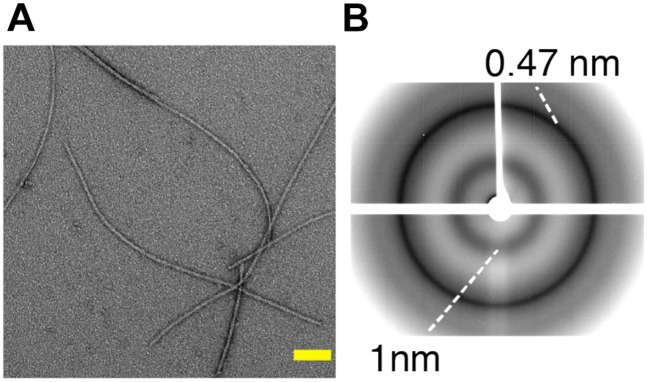
**(A)** Negatively stained electron micrographs of the C-terminal fragment TDP-35 involved in amyotrophic lateral sclerosis, showing fibrillar aggregates. Scale bar (in yellow) is 100 nm. **(B)** X-ray diffraction pattern of TDP-35 aggregates showing characteristic cross-β diffraction pattern in amyloid fibrils, with reflections at 4.7 and 10 Å corresponding to the inter-strand and inter-sheet spacing respectively. Adapted from Shenoy et al. (2019).

The effective functioning of proteins solely entrusts on its proper 3D folding, which is controlled by complex cellular mechanisms. But these complex mechanisms are prone to errors resulting in misfolding and aggregation of native proteins into ordered aggregates termed amyloids (Chiti and Dobson, 2006). These extracellular aggregates are proteinaceous assemblies rich in β-sheet secondary structures and capable of acting as templates that convert soluble forms of native proteins to adopt the same conformation, resulting in insoluble amyloid deposits. The fibrillar aggregates are observed as elongated, unbranched protofilaments typically of ∼5-10 nanometers in width and a few micrometers in length (Sunde and Blake, 1997). As an illustration, Figure 2A shows the fibrillar aggregates formed by the C-terminal fragment of TDP-43, called TDP-35 (Shenoy et al., 2019), associated with amyotrophic lateral sclerosis (Neumann et al., 2006). The site and nature of amyloid deposition correlate with many neurodegenerative diseases like AD, Parkinson’s disease, Creutzfeldt-Jakob disease (CJD) and numerous other prion-like diseases (Selkoe, 2000; Goedert, 2001b; Spillantini, 2001; Beyer and Ariza, 2007; Knowles et al., 2014). The amyloid-related pathology can be associated with two deleterious mechanisms: (i) the inability of the amyloid-forming protein to perform its native function, termed as ‘loss of function mechanism’ and (ii) the amyloid deposit itself is to the cellular metabolism, referred as ‘toxic gain of function’ (Winklhofer et al., 2008).

Regardless of the difference in their primary sequence, most amyloids share a common structural characteristic called cross-β, which gives rise to a characteristic X-ray diffraction pattern (Geddes et al., 1968). A generic cross-β fold consists of an orderly arrangement of β-sheets, in which the hydrogen bonding directions run parallel and the β-strands are orthogonal to the fiber axis, as in rungs of a ladder (Geddes et al., 1968). This continuous packing of cross β-sheets generates a unique X-ray diffraction pattern with a meridional reflection at ∼4.7 Å and an equatorial diffraction signal at ∼6–12 Å corresponding to inter-strand and inter-sheet spacing, respectively (Eanes and Glenner, 1968; Geddes et al., 1968; Makin et al., 2005). Figure 2B illustrates the cross-β diffraction pattern obtained on amyloid fibrils of TDP-35. Several high-resolution structures of 3D microcrystals of short peptide segments derived from pathogenic amyloid fibrils (e.g., Aβ, tau, α-synuclein, TDP-43) have been reported (Nelson et al., 2005; Wiltzius et al., 2009; Guenther et al., 2018). These studies have highlighted a specific structural feature termed ‘steric zipper,’ in which the adjacent β-sheets are tightly held together with facing side chains interdigitated via an interface devoid of water (Sawaya et al., 2007). Although cross-β patterns are a diagnostic hallmark of amyloid, any structural information leading to high-resolution structures is limited due to the difficulty in growing highly ordered crystals from amyloid fibrils due to their polymorphic nature, and to provide the tertiary and quaternary arrangement that can be very different among amyloid aggregates.

### A Long Quest for the High-Resolution Structure of Pathological Amyloids

Transmissible spongiform encephalopathies (TSEs) – such as Creutzfeldt–Jakob disease, fatal familial insomnia and kuru - have been associated with the “protein-only” hypothesis, specifying that the disease pathogenesis is triggered by the conformational transition between the native cellular fold of a protein (PrP^*C*^) to infectious aggregates (PrP^*Sc*^), enriched in β-sheet secondary structure (Aguzzi and Polymenidou, 2004). Many structural studies (Wille and Requena, 2018) using notably solution and solid-state NMR spectroscopy have been carried out to obtain structural details about the prion architecture and the molecular events associated with the prion aggregation. In the late 90s, Riek et al. (1996, 1997) uncovered the structural fold of the recombinant truncated form of mouse and murine PrP using solution NMR, the structural features being conserved with other mammalian PrP orthologs (Lysek et al., 2005). The first X-ray structure of recombinant human prion PrP^*C*^ (Knaus et al., 2001) and several variants (Lee et al., 2010) revealed the presence of a domain-swapped dimer. Because of its insolubility and non-crystallinity, PrP^*Sc*^ aggregates constitute challenging targets for solution NMR or X-ray crystallography. Several hypothetical structural models have been proposed on the basis of high-resolution structure of truncated protein variants and low-resolution information about the structural conversion. A parallel left-handed β-helical fold (Govaerts et al., 2004) was proposed based on electron microscopy on the N-terminally truncated PrP 27–30 and the mini-prion PrP^*Sc*^106 (Wille et al., 2002). DeMarco and Daggett (2004) presented a β-spiral model using molecular dynamics simulations. Based on hydrogen-deuterium exchange, Lu et al. (2007) and Smirnovas et al. (2011) proposed a model exhibiting a complete refolding of the protein into non-α-helical but mostly β-sheet structure. Wille and coworkers reported cryo-electron microscopy data supporting a four-rung β-solenoid structure (Vázquez-Fernández et al., 2016). A refined model was recently generated (Spagnolli et al., 2019) and represents to-date the most plausible structural features of PrP^*Sc*^. Interestingly, the four-rung β-solenoid structure appeared as stable as the naturally occurring β-solenoid of the fungal prion HET-s (Wasmer et al., 2008) described later in this review. Many SSNMR studies have focused on the Y145Stop prion variant (PrP23-144) by the Jaroniec group to decipher its secondary structure (Helmus et al., 2008), conformational flexibility (Helmus et al., 2010), intermolecular packing (Helmus et al., 2011), and polymorphism (Jones et al., 2011). In 2017, a species-dependent behavior was observed for Y145Stop prion amyloid fibrils by comparing SSNMR spectra from human, mouse and Syrian hamster fibrils (Theint et al., 2017). Liang et al. proposed a high-resolution structural model of PrP^*sc*^ from in vitro recombinant protein expression using cryo-electron microscopy, and the structure comprises an amyloid core spanning residues 170–229 (Wang et al., 2020). Kraus et al. (2021) recently uncovered a larger rigid core for purified PrP fibrils from hamster brains, composed of residues 95–227, with a structural arrangement that differs from structural models of non-infectious fibrils. Despite progress in studying PrP^*Sc*^ recombinant protein and brain-derived structures, more atomic scale information on fibrils from various mammalian specimens will be required to understand how the fibril structure might be related to prion strains and pathogenicity.

Protein misfolding and aggregation is also associated with other neurodegenerative diseases (Stefani and Dobson, 2003). Formation of senile plaques and neurofibrillary tangles (NFTs) composed of amyloid-β (Aβ) and tau, respectively, is considered to be the biological hallmark of AD (Rasool and Selkoe, 1984). Aβ1–40 and Aβ1–42 originating from the proteolytic fragmentation of the amyloid precursor protein (APP), are the most prominent forms of amyloid fibrils found in amyloid plaques of Alzheimer’s patients (Haass and Selkoe, 1993). The aggregation of microtubule-associated protein Tau is associated with a broad range of brain diseases generally termed tauopathies, which includes AD, progressive supranuclear palsy, argyrophilic grain disease, corticobasal degeneration and Pick’s disease (Lee et al., 2001; Avila et al., 2004). Similarly, deposition of insoluble aggregates of intrinsically disordered protein α-synuclein in Lewy bodies and Lewy neurites is involved in etiologies of Lewy body-related disorders including Parkinson’s disease, dementia with Lewy bodies (DLB) and other neurodegenerative conditions, termed synucleinopathies (Goedert, 2001a; Lippa et al., 2007; Breydo et al., 2012).

**FIGURE 3:**
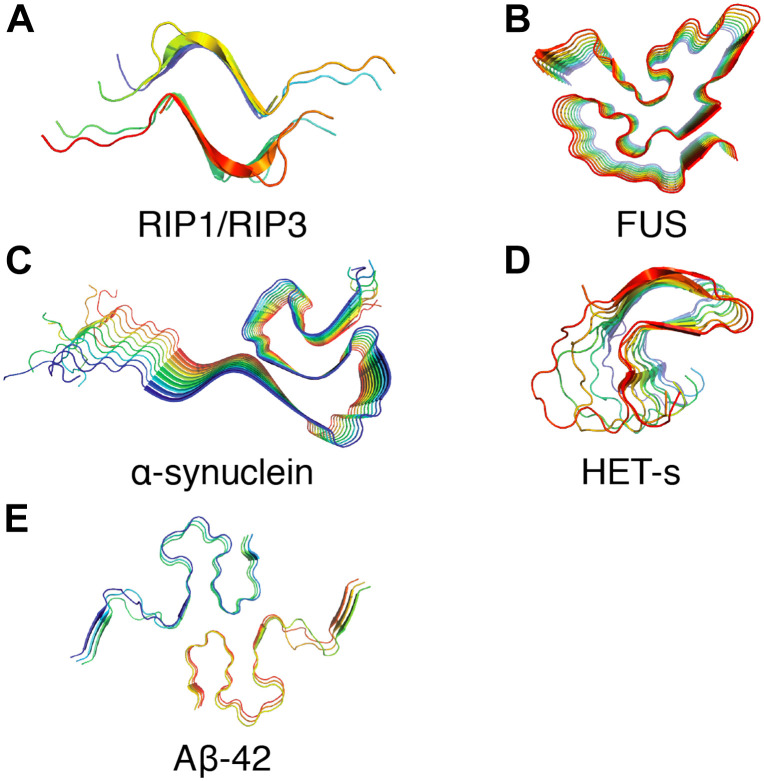
Examples of amyloid fibrils atomic structures determined by solid-state NMR spectroscopy. **(A)** Structure of the human RIPK1-RIPK3 necrosome1 (PDB ID: 5V7Z) (Mompeán et al., 2018). **(B)** Structure of the RNA-binding protein FUS (PDB ID: 5W3N) (Murray et al., 2017). **(C)** Structure of α-synuclein fibrils (PDB ID: 2N0A) (Tuttle et al., 2016). **(D)** Structure of fungal prion HET-s (218–289) (PDB ID: 2RNM) (Wasmer et al., 2008). **(E)** Structure of Aβ42 fibrils (PDB ID: 2NAO) (Wälti et al., 2016).

The non-crystalline and insoluble properties of amyloid fibrils have hindered the path to establishing atomic resolution structures by conventional techniques such as X-ray crystallography or solution NMR. Recently, SSNMR spectroscopy and cryo-electron microscopy approaches have been extensively used to investigate atomic resolution structures of such pathogenic proteins in their fibrillar form. Figure 3 presents several amyloid fibril structures solved at atomic resolution by SSNMR. Based on pioneering work from Tycko group, structures of Aβ1–40 have been proposed by SSNMR with a common motif with two β-sheets flanged by a loop (Petkova et al., 2002, 2006; Paravastu et al., 2008; Lu et al., 2013). Additionally, structural models of Aβ1–40 fibrils bearing the pathologically relevant Iowa and Osaka mutations were proposed based on SSNMR data (Qiang et al., 2012; Schütz et al., 2015; Sgourakis et al., 2015). Using Aβ1–40 fibrils seeded from brain extracts from two AD patients, Lu et al. (2013) have observed two different polymorphs by SSNMR and one of the polymorphs revealed a threefold structural symmetry similar to *in vitro* prepared fibrils. Aβ1–40 and Aβ1–42 fibril conformation were also assessed in various Alzheimer’s disease clinical subtypes by SSNMR (Qiang et al., 2017). Early studies on Aβ1–42 proposed a similar “U”-shaped model with residues 18–26 and residues 31–42 forming the β-strands connected by a loop and stabilized by salt bridges between residues D23–K28 (Lührs et al., 2005). Recently, atomic resolution structures from SSNMR data of Aβ1–42 suggested a “double horseshoe” or an “S”-shaped chain arrangement comprising three β-strands (Colvin et al., 2016; Wälti et al., 2016). Aβ1–42 fibrils composed of two intertwined protofilaments determined by cryo-electron microscopy suggested an overall “LS”-shaped topology of individual subunit (Gremer et al., 2017).

In the case of Parkinson’s disease-associated α-synuclein, early studies by Heise and Baldus demonstrated the use of ^13^C and ^15^N SSNMR chemical shifts to decipher the presence of two different α-synuclein conformations correlated with the fibril morphology (Heise et al., 2005). Extensive studies by the Rienstra group (Kloepper et al., 2007; Comellas et al., 2011) led to the establishment of an atomic resolution structure by SSNMR using a large set of distance restraints. The structure demonstrates a Greek-key topology with the core residues arranged in a parallel in-register β-sheet arrangement (Tuttle et al., 2016). Recent studies by cryo-electron microscopy revealed various inter-fibrillar conformation and quaternary arrangement depending the source of brain-derived samples (Guerrero-Ferreira et al., 2018; Schweighauser et al., 2020). Those fibrillar architectures resulting from brain extraction can be distinct from *in vitro* fibrils obtained after recombinant α-synuclein expression in *Escherichia coli*, highlighting that a cautious approach should be taken in considering fibrillar structure resulting from *in vitro* aggregation. Systemic amyloidosis are fatal diseases and they have been associated with protein misfolding and the deposit of amyloid fibrils. Fibrils from systemic AA (Liberta et al., 2019), AL (Liberta et al., 2019; Swuec et al., 2019), and ATTR (Schmidt et al., 2019) are now available from cryo-EM studies. In this context, recent work on serum amyloid A (SAA) (Bansal et al., 2021) exemplified possible structural differences between fibrils obtained from recombinant protein aggregation (*in vitro*) and fibrils purified from tissues.

## Functional Prions Emerge as *Trans*-Kingdom Entities

### The HET-s Prion Paradigm

The [Het-s] prion from the mold *Podospora anserina* controls a programmed cell death (PCD) reaction termed heterokaryon incompatibility (HI) (Saupe, 2011; Paoletti, 2016; Daskalov et al., 2017). HI occurs in the fusion cells between genetically distinct fungal individuals and prevents the formation of a common syncytial network. The reaction limits the spread of mycoviruses and prevents genome exploitation, acting as a fungal specific defense mechanism (van Diepeningen et al., 1997; Zhang and Nuss, 2016). In that regard, the [Het-s] prion controlling the HI cell-death reaction is a functional prion and is highly prevalent in wild isolates of *P. anserina (Wickner, 1997; Debets et al., 2012)*.

**FIGURE 4:**
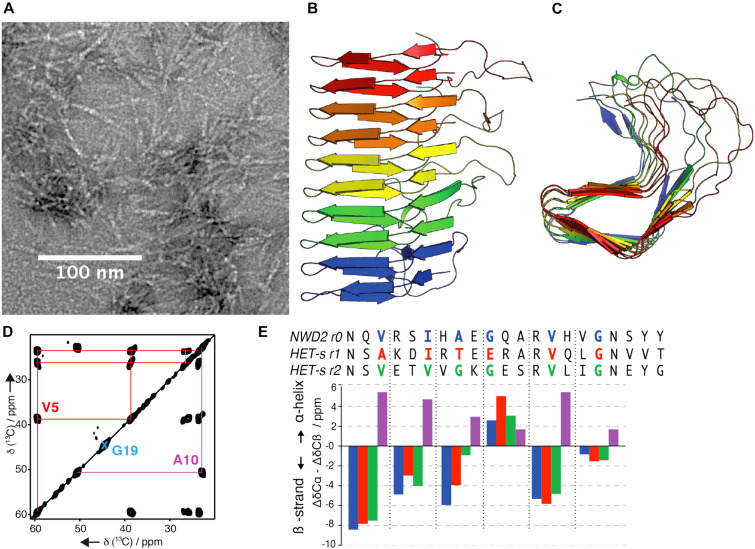
**(A)** Electron micrograph of HET-s PFD fibrils, scale bar is 100 nm. **(B,C)** Solid-state NMR structure of HET-s prion-forming domain, (Wasmer et al., 2008), front view **(B)** and top view **(C)**. **(D)** Solid-state NMR experiments on selectively labeled NWD2 (3–23) amyloid fibrils to determine the secondary structure encoded in the ^13^C chemical shift values. **(E)** Comparison of the secondary structure of NWD2 (3–23) with the two PFD repeats of HET-s. Negative or positive values indicate β-strand or α-helix conformation respectively for the color-coded amino acids residues shown in the sequences above the bars (Daskalov et al., 2014). Pink bars are showing hypothetical chemical shifts for each of the colored corresponding residues of NWD2 in α-helical conformation.

The [Het-s] prion is based on a 289 amino acids protein, termed HET-s (small “s”) (Coustou et al., 1997). The HET-s protein can exist in a monomeric state termed [Het-s^∗^] (small “s star”) or the prion aggregates [Het-s]. Strains of the *het-s* genotype are exclusively prion-free or prion-infected. Only prion-infected [Het-s] strains produce HI cell-death with strains from the *het-S* (large “S”) genotype (Saupe, 2011). Thus, the HI reaction is defined by an allelic incompatibility between the *het-s* and *het-S* alleles (Turcq et al., 1991). The two allelic variants – HET-s and HET-S – differ at only 13 amino acids positions and share the same domain architecture with an N-terminal globular HeLo domain and an unstructured C-terminal prion-forming domain (PFD) (Coustou et al., 1999; Balguerie et al., 2003). The allelic specificity is carried by the HeLo domain and it has been established that a mutation has occurred in the HET-S variant, which has hindered the cytotoxic activity of the HeLo domain, hence resulting in the genesis of HET-s, the prion protein (Daskalov and Saupe, 2015). The PFD domains – or HET-s/S(218–289) – of both allelic variants are interchangeable (Balguerie et al., 2003). The structure of the prion amyloid fibers (Figure 4A) formed by HET-s(218–289) has been solved by SSNMR (Figures 4B,C) by Wasmer et al. (2008) and has become a reference experimental model for the study of the fundamental properties of prion amyloids.

The PFD consists of two 21 amino acids pseudo-repeats (termed R1 and R2) connected by a flexible 15 amino acid long loop. The repeats are alternately stacked along the axis of the β-solenoid, each contributing with four β-strands per rung. The resulting β-sheets delimit a highly hydrophobic triangular β-solenoid core. The core is tightly packed with predominantly hydrophobic residues while polar and charged residues are found on the exterior, solvent-facing side of the amyloid (Wasmer et al., 2008). We have identified a homologous region to the PFD repeats (R1/R2) at the extreme N-terminal part of a protein named NWD2 and encoded by the gene adjacent to *het-S* (Daskalov et al., 2012). On the basis of SSNMR data (Figures 4D,E), we proposed that the NWD2 segment 3–23 adopts the same structure as a single PFD repeat of HET-s (Daskalov et al., 2015b). NWD2 is a fungal NOD-like receptor (NLR). NLRs are a family of intracellular receptors involved in innate immunity in plants and metazoans (Proell et al., 2008; Jones et al., 2016). While the PFD of HET-S/s contains two pseudo-repeats, NWD2 contains only one repeat, termed R0 (Daskalov et al., 2012). It is proposed, based on the accumulated experimental evidence, that the oligomerization of NWD2 brings the R0 motifs of different NWD2 molecules in proximity so that these motifs cooperatively adopt a HET-S-like amyloid fold and subsequently template the PFD of HET-S to trigger the cytotoxic activity of the HeLo domain (Seuring et al., 2012; Daskalov et al., 2015b; Riek and Saupe, 2016). Thus, the amyloid fold adopted by HET-S(218–289) is an integral component of a signal transduction mechanism (Daskalov et al., 2015b; Riek and Saupe, 2016). While the HI incompatibility between the *het-s*/*het-S* alleles is present only in *P. anserina*, gene clusters of *het-S*/*nwd2* are present in the genomes of dozens of fungal species (Daskalov et al., 2012; Saupe and Daskalov, 2012). The widespread and diversity of amyloid prion domains underscores the evolutionary success of this class of signaling modules in fungi. Furthermore, we have uncovered a large variety of signaling amyloids with prion properties operating in analogous NLR-dependent signaling cascades (Daskalov et al., 2015a; Daskalov, 2016; Loquet and Saupe, 2017). In particular, the fungal PP motif (Daskalov et al., 2016) shows sequence similarity to the RIP1/RIP3 necrosome in humans. Preliminary SSNMR analysis of the recombinant PP motif revealed the formation of well-ordered amyloid fibrils rich in β-sheet structure (Daskalov et al., 2016). A structural model of RIP1-RIP3 has been proposed based on SSNMR structural information (Mompeán et al., 2018), recently complemented by the structure of RIPK3 solved by cryo-EM in combination with SSNMR (Wu et al., 2021), and it remains to be solved if the sequence similarity between fungal and mammals necroptotic amyloid motifs translate into structure similarity.

### Functional Amyloids Are Multipurpose Entities

The unique properties of amyloid assemblies reside in their very loose sequence-to-fold relationship, enabling a very high variability of amyloidogenic sequences that are spread in multiple biological systems. Prokaryotic and eukaryotic cells take advantage of the protease and detergent resistance properties characterizing amyloid fibrils as a structural component in the regulation of chemical reactions or gene expression within the cell or in the storage of peptides. For instance, bacteria utilize amyloid pathways to increase the half-life and the spread of biofilms in human hosts or medical devices by reinforcing the robustness of their colonies. In *Escherichia coli*, Curli proteins CsgA and CsgB are secreted through the outer membrane in a monomeric state to form a membrane-mediated mature fibril at the cell surface in order to protect the integrity of the bacterial colonies and allow for cell adhesion (Barnhart and Chapman, 2006). CsgA and CsgB monomers diffuse across the membrane using a complex machinery composed of CsgG/CsgE multimers forming a pore embedded in the bacterial outer membrane (Nenninger et al., 2011; Goyal et al., 2014). The polymerization process of CsgA is tightly regulated by accessory proteins such as CsgC, which maintain monomeric form until they are properly addressed to the extracellular compartment (Evans et al., 2015).

In *Pseudomonas* species, amyloid assemblies mainly composed of FapC subunits are formed at the cell surface to increase the hydrophobicity of the biofilm and attach to abiotic areas (Dueholm et al., 2013). This confers an extreme robustness to *Pseudomonas* biofilms causing severe infections in immunosuppressed patients (Moser et al., 2017). The amyloidogenic Fap complex (for Functional Amyloid in Pseudomonas) is composed of six different proteins (FapA-FapF), which are mainly implicated in the stabilization of Fap subunits monomeric state, the transmembrane transport and the formation of native assembly (Dueholm et al., 2010). Similarly to Curli, monomeric subunits are secreted outside of the cell through a membrane channel and converted to a mature fibril by the nucleator protein FapB (Dueholm et al., 2013). Multiple proteins with amyloid-forming properties have been identified in different bacterial taxa such as MTP in *Mycobacterium* (Alteri et al., 2007), chaplins in *Streptomyces* (Elliot et al., 2003), P1 in *Streptococcus* (Heim et al., 2015) or PSM (Tayeb-Fligelman et al., 2017), and Bap (Taglialegna et al., 2016) in *Staphylococcus*. A similar function is also observed in eukaryotic cells such as fungi whose spores surface is surrounded by a hydrophobic layer mainly composed of amyloid fibrils (Bayry et al., 2012). The role of these fibrils – referred as hydrophobins – is observed in the adhesion to the cell surfaces and confers non-immunogenic properties to the spores, which become a serious threat for immunocompromised patients (Talbot et al., 1996; Aimanianda et al., 2009).

For most of these functional amyloids, the high-resolution structure of the aggregated state is unknown. SSNMR has been employed to deliver crucial information on the secondary structure and intermolecular packing of functional amyloids, e.g., on the supramolecular arrangement of CsgA and CsgB (Shewmaker et al., 2009), the molecular conformation of fungal hydrophobins (Morris et al., 2012), the structural characterization of the functional amyloid Orb2 involved in memory formation (Cervantes et al., 2016), the functional β-endorphin fibrils (Nespovitaya et al., 2016) or the gas vesicle protein A (Bayro et al., 2012). In *Bacillus* species, amyloid fibrils formed by the protein TasA have been linked to the structural integrity of the biofilm extracellular matrix (Romero et al., 2010). SSNMR has offered a method of choice to investigate such heterogeneous fibrils at atomic level. Two independent SSNMR studies have provided evidence on the amyloid nature of TasA biofilm filaments (Diehl et al., 2018; El Mammeri et al., 2019).

Amyloid aggregates have also been highlighted as a major component of the fish and insect eggshell, named chorion. This natural envelope plays a crucial role both physiological and protective in the development of the oocyte and the survival of the embryo (Hamodrakas et al., 2004). Although the chorion is composed of hundreds of different proteins, numerous biophysical studies applied to these supramolecular structures led to the conclusion that it adopts structural signature compatible with the well-established amyloid signature (Hamodrakas et al., 1982; Tsiolaki et al., 2018). The yeast *Saccharomyces cerevisiae* takes advantage of the prion-forming properties to change their phenotypes. For example, Sup35p and Ure2p are two proteins involved in the termination of the translation (Stansfield et al., 1995) and the regulation of the nitrogen metabolism (Wickner, 1996), respectively. When good nitrogen sources are present in the environment, Ure2p soluble homodimers block the action of transcription regulators involved in the assimilation of poor nitrogen sources (Lacroute, 1971). When Ure2p aggregates are formed *in vivo*, their stability is enhanced and they no longer bind properly the transcription factors, leading subsequently to the expression of genes used for poor nitrogen sources and a slow growth (Edskes et al., 1999; Brachmann et al., 2005; Shewmaker et al., 2007). The aggregation of Sup35p results in a decrease in the number of functional proteins leading to an increased rate of translational read-through of stop codons. Depending on the growth conditions, aggregation of Sup35p monomers leads to different phenotypes, such as a better tolerance to environmental stresses (Eaglestone et al., 1999; True and Lindquist, 2000) or to the cell death by sequestrating other translation termination factors into Sup35p aggregates (McGlinchey et al., 2011a). Ure2p and Sup35 have been extensively investigated by SSNMR techniques. Chan et al. (2005), Baxa et al. (2007), and Kryndushkin et al. (2011) proposed NMR-based structural models of the prion domain of Ure2p (1–89). Loquet et al. (2009) showed that the globular domain of Ure2p (70–354) could keep its tertiary fold and structural rigidity in the amyloid assembly under specific conditions, as revealed by SSNMR chemical shift values conserved between the crystalline form of Ure2p (70–354) and full-length Ure2p fibrils. It pointed out the variable conformational behavior concerning the non-amyloid domains during the aggregation process of such prions. Wickner and Tycko pioneered studies on Sup35 amyloid assemblies (Shewmaker et al., 2006; Gorkovskiy et al., 2014) using SSNMR. Luckgei et al. (2013) demonstrated by SSNMR that full-length Sup35 and Sup35 (1–253) fibrils have different conformation, stressing the fact that amyloid domains might adopt different structures in isolation or in the context of full-length protein assembly.

In mammals, PMEL fibrils have emerged as the first human functional amyloid described in the literature (Fowler et al., 2006). Multiple intermediates formed during the synthesis pathway of melanin are often highly toxic to the cell (Graham et al., 1978), which requires the use of protective strategies to sequestrate these compounds. Pmel17 is a membrane-associated protein, which produces amyloid fibrils after proteolytic cleavage (Watt et al., 2013; Bissig et al., 2016). These fibrils function as a protective surface to isolate toxic intermediates and enhance melanin synthesis (Fowler et al., 2006). SSNMR studies by the group of Wickner on recombinant PMEL fibrils (McGlinchey et al., 2009), combined with mass-per-length measurements (McGlinchey et al., 2011b) provided structural evidence of an in-register parallel β-sheet arrangement with two Pmel17 domain per fibril layer.

## High-Resolution Protein Structure Determination by Solid-State NMR

### Solid-State NMR and Magic-Angle Spinning

Solid-state NMR is a spectroscopic technique widely used to characterize solid-like (bio)materials (McDowell and Schaefer, 1996; McDermott, 2009; Weingarth and Baldus, 2013). Insoluble biological samples such as aggregates, oligomeric species and fibrillar assemblies contain strong anisotropic and dipolar interactions, which have a spatial dependency. Although these interactions can be crucial to extract specific information about the dynamics of the studied protein system (Bechinger et al., 2011; Vugmeyster and Ostrovsky, 2017), they heavily broaden the line-width of resonances in SSNMR spectra. As a result, many resonances overlap, hampering further analysis at site-specific resolution. In solution NMR, anisotropic interactions are averaged out by rapid molecular tumbling motions, which lead to sharp resonances. However, the increased size of the molecule is slowing the tumbling motion, reducing the transverse relaxation times and hence increases the line-width of NMR resonances. This is one of the limiting factors in the study of very large molecules by solution NMR. Because the strength of anisotropic interactions is orientation-dependent, the mechanical alignment of the sample at a particular angle (called the magic angle) with respect to the static magnetic field and spinning around the tilted axis averages out these anisotropic interactions, resulting in more resolved peaks and enabling so-called high-resolution magic-angle spinning (MAS) solid-state NMR studies (Andrew et al., 1958).

**FIGURE 5:**
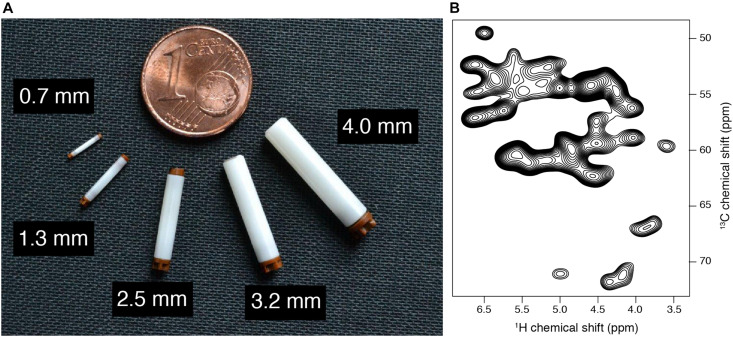
**(A)** Various solid-state NMR rotors, diameters are highlighted. **(B)** 2D CP hCH spectrum of Ha-Ca region in uniformly labeled HET-s (218–289) fibrils, acquired at 100 kHz MAS on a 18.8 T magnet.

As a consequence, many biological systems usually challenging for crystallography and solution NMR constitute nowadays targets of choice for SSNMR investigations. In order to efficiently apply SSNMR spectroscopy for high-resolution studies, the rotation around the magic angle needs to be experimentally combined with spin decoupling (Mote et al., 2016) and recoupling techniques (Ladizhansky, 2009; Demers et al., 2011). Technological developments of NMR probes allowing fast (30–40 kHz MAS) (Ishii and Tycko, 2000; Ishii et al., 2001; Ernst et al., 2004; Zhou et al., 2007; Bertini et al., 2010) to ultra fast spinning (>60 kHz MAS) (Agarwal et al., 2014; Wang et al., 2015a, b; Andreas et al., 2016), have allowed the implementation of various kind of spin manipulation techniques, leading to the extraction of different structural features. Figure 5A presents various rotor sizes used for biomolecular studies, ranging from 4 mm diameter rotor (10–100 mg sample quantity, routinely used at 11 kHz MAS) to 0.7 mm diameter rotor (0.5–1 mg sample quantity, routinely used at 100 kHz MAS, as illustrated in Figure 5B for HET-s amyloid fibrils). Nowadays, many semi-automatic setups are available as state-of-the-art for commercial SSNMR probes, which are well adapted for the needs of structural biology research (Martin et al., 2019). These technological developments, combined with the increased number of available high-field NMR magnets and advanced protein labeling techniques have considerably improved the analytical capability of SSNMR (Lacabanne et al., 2019). These approaches have been used to unlock structures of various types of biological systems. Numerous examples of biomolecular systems solved by SSNMR could be found in more dedicated reviews here (Baker and Baldus, 2014; van der Wel, 2017; Demers et al., 2018; Loquet et al., 2018a; Mandala et al., 2018; Marchanka and Carlomagno, 2019).

In the early 2000’s, the three-dimensional structures of small microcrystalline proteins (Castellani et al., 2002; Zech et al., 2005), short peptides (Rienstra et al., 2002), and the first atomic resolution model of an amyloid fibril (Wasmer et al., 2008) have paved the way to high-resolution structural investigation of more complex protein complexes, including pathological amyloid aggregates (Schütz et al., 2015; Colvin et al., 2016; Tuttle et al., 2016; Wälti et al., 2016). The horizon of structural studies has also been extended to filaments (Loquet et al., 2012; Vasa et al., 2015), viral capsids assembly (Andreas et al., 2016; Lu et al., 2020), and membrane proteins inserted in the lipid bilayers (Cady et al., 2010; Wang et al., 2013).

### Amyloid Structure Determination by Solid-State NMR and Perspectives on Integrative Approaches

High-resolution structure determination of amyloids depends on the collection of appropriate SSNMR data sets. Contrary to X-ray crystallography or electron microscopy, NMR spectroscopy does not provide direct information on the 3D structures, i.e., spatial position of atoms, but rather indirect measurements such as local conformations (e.g., from chemical shifts) or atom proximities in space. For instance, in carbon NMR experiments such as PDSD (Proton Driven Spin diffusion) or DARR (Dipolar Assisted Rotational Resonance) magnetization is transferred between ^13^C nuclei that are close in space. From such spectra, it is then possible to collect cross-peaks correlating ^13^C atoms within the same residue or between different residues (up to 8–10 Å) by varying the experimental NMR set up (Castellani et al., 2002). Identified proximities through cross-peak assignment are converted into spatial restraints, similarly to solution NMR methods. Yet, computational methods are required to generate molecular conformations satisfying these restraints. The molecular structures are calculated with dedicated software such as CYANA (Güntert et al., 1997), ARIA (Bardiaux et al., 2012), UNIO (Guerry and Herrmann, 2012), Xplor-NIH (Schwieters et al., 2003), or Rosetta (Shen et al., 2008).

Owing to the repetitive nature of amyloid fibers where monomers are stacked along the main fiber axis, through-space SSNMR correlations may arise from intra-monomeric (i.e., with the same protein monomer), and/or inter-monomeric proximities (i.e., between neighboring protein monomers). Hence, structure determination of amyloid structures generally benefits from a three-step labeling strategy in order to collect intra- and inter-molecular restraints. First, data acquisition from a ^13^C and ^15^N uniformly labeled sample provides information from both intra- and inter-molecular proximities without distinction, providing highly ambiguous resonance assignments. Second, a 1:x (with *x* = 3–5) diluted sample of ^13^C/^15^N labeled monomers mixed with unlabeled (^13^C/^15^N at natural abundance) monomers greatly reduce the inter-monomeric contribution, thus allowing the unambiguous gathering of intra-molecular interactions (Wasmer et al., 2008). Finally, the use 1:1 mixtures of ^13^C labeled and ^15^N labeled samples (Etzkorn et al., 2004) or 1:1 mixtures of specifically ^13^C labeled samples (Loquet et al., 2010) is determinant to identify and collect unambiguous inter-molecular proximities to establish the supramolecular stacking of amyloid fibrils.

Assignments of cross-peaks to individual atom pairs remain, however, potentially ambiguous due to insufficient spectral resolution of SSNMR experiment, compared to solution NMR. Furthermore, in the case of amyloid fibrils made of two or more protofilaments, inter-molecular correlations may be originating from axial contacts (along the fibril), or contacts that are made with the neighboring protofilaments. Those issues are generally tackled by starting with subsets of spectrally unambiguous restraints to determine a reasonable approximate fold of the protein. Subsequent iterations of the calculation will then use more restraints that are found compatible with the initial fold, thus solving ambiguities along the way. Resolving spectral and symmetry-related ambiguities is a tedious task. Similar to solution NMR, automated approaches, as implemented in the CYANA (Güntert et al., 1997) and ARIA (Bardiaux et al., 2012) software, can streamline the assignment process using an iterative procedure to reduce ambiguities in the restraints while producing high-quality structures ensembles (Loquet et al., 2008; Manolikas et al., 2008). The sets of distance restraints are generally supplemented by angular restraints on the dihedral backbone angles, predicted from secondary chemical shifts with empirical models like TALOS+ (Shen et al., 2009). Symmetry is generally enforced during computation to maintain a similar fold of monomers along the fibril (Nilges, 1993). Inter-molecular distances are also replicated along the axis of the fibril to be applied on each layer of the fibril.

Stacking properties are complementarily observed with other experimental techniques to help fully characterize amyloid filaments. For example, X-ray diffraction reveals the cross-β architecture and stacking distances of amyloid fibrils (Sunde and Blake, 1997). A very representative diffraction pattern allows measuring the inter-sheet and inter-strand distances. Mass-per-unit-length (MPL) parameters can be obtained by scanning transmission electron microscopy (STEM) measurements (Goldsbury et al., 2011). MPL data provide key information about the number of monomers in a β-sheet layer (∼0.47 nm) thus allowing to decipher the composition of fibril layers, like in n-fold Aβ filaments or the two windings of the HET-s β-solenoid (Chen et al., 2009).

As an increasing list of complex amyloid fibril arrangements is being studied, integrative hybrid approaches for structural determination that combine data from various sources are becoming more relevant (Cuniasse et al., 2017). The recent emergence of cryo-EM at high resolution has provided a powerful approach to solve the structure of homogeneous fibrillar amyloid preparations at high resolution (Röder et al., 2019; Schmidt et al., 2019). One key advantage of the technique lies in its ability to analyze amyloid fibrils extracted *ex vivo*, this procedure being extremely arduous for SSNMR since this type of material cannot be isotopically labeled. Apart from atomic resolution structure determination, EM is often useful at lower resolution to determine the overall shape, architecture and symmetry parameters of amyloid fibrils, while SSNMR can complement this structure determination process with local atomic interactions (Gremer et al., 2017). Polymorphism in amyloid samples can also hinder the determination of high-resolution structures of amyloid fibrils by SSNMR. Close et al. (2018) have proposed a detailed rational analysis on amyloid structural polymorphism based on high-resolution cryo-EM and SSNMR measurements. In this context, cryo-EM can help in alleviating assignment ambiguities while SSNMR can help initial model building in cryo-EM densities, as shown for TTR(105-115) peptide amyloid fibrils (Fitzpatrick et al., 2013) and more recently for full-length α-synuclein (Guerrero-Ferreira et al., 2019). In view of the tremendous benefit of combining STEM, cryo-EM and SSNMR for structure determination of helical filaments in the last decade (Loquet et al., 2012; Demers et al., 2014; Habenstein et al., 2015; Sborgi et al., 2015), such a hybrid approach will undoubtedly help to resolve more complex and composite amyloid structures in the future.

### New SSNMR Approaches and the Emergence of ^1^H Detection and High-Sensitivity Methods

**FIGURE 6:**
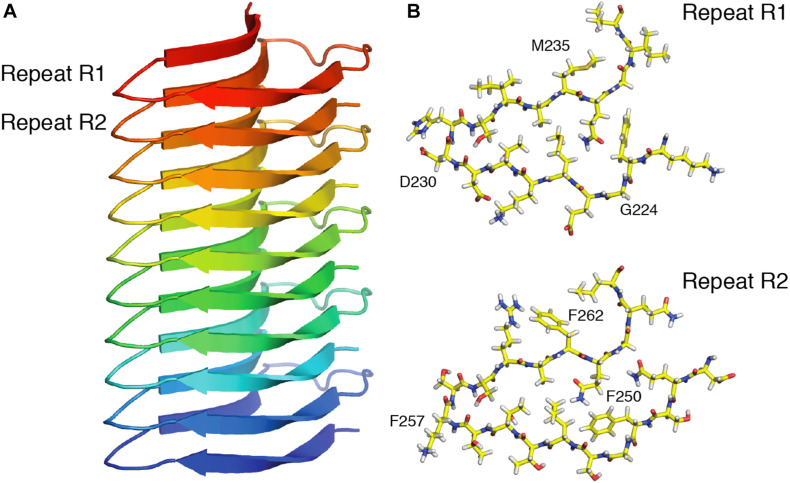
High resolution SSNMR structure of the HELLF prion amyloid solved from ^1^H-^1^H distance restraints. **(A)** Front view, showing 5 HELLF subunits stacked in a cross-β amyloid fold. **(B)** Top view of the two amyloid pseudo-repeats (R1 and R2). Adapted from Daskalov et al. (2021).

High sensitivity ^13^C- and ^15^N-detected multi-dimensional SSNMR experiments are usually obtained at moderate MAS frequencies (10–20 kHz). In order to achieve good sensitivity, ∼10–50 mg of labeled material is necessary to carry out 2D and 3D multidimensional experiments. The use of higher gyromagnetic ratio ^1^H nucleus instead of ^13^C and ^15^N nuclei can compensate the low sensitivity. ^1^H detection would lead to a sensitivity increase of 8-fold compared to ^13^C detection (Ishii and Tycko, 2000). However, due to the presence of strong (up to 50 kHz) ^1^H-^1^H dipole couplings, which leads to shorter transverse relaxation times T_2_’, the ^1^H line-width is significantly broader compared to ^13^C. A valid strategy is to replace the majority of ^1^H nuclei with ^2^H nuclei (Reif, 2012), thus reducing the strong network of dipolar couplings. At moderate MAS frequencies (around 20 kHz), high-resolution 2D and 3D experiments can be achieved (Linser et al., 2011; Zhou et al., 2012). Although the ^1^H line-width was significantly decreased, this approach implies higher sample preparation costs due to the use of D_2_O in labeled media. Another approach to reduce the ^1^H line-widths is to increase the sample spinning frequencies. Recent advances in the technology development of SSNMR probes enabled to tremendously increase the rotation frequency, ∼140–170 kHz being the fastest one published up to date (Schledorn et al., 2020), and requiring less than 0.5 mg of sample. It was demonstrated with the increasing MAS rates that ^1^H T_2_’ coherence times are increasing as well with a linear quadratic dependency (Sternberg et al., 2018; Penzel et al., 2019). As a result, homogenous contributions to the experimental line-widths are reduced. From the measured data, it has been extrapolated that MAS frequencies of around 300 kHz (for deuterated samples) (Xue et al., 2018; Penzel et al., 2019) and 1000 kHz (for fully protonated) proteins (Penzel et al., 2019) would be necessary in order to reach line-widths similar to liquid-state NMR. Still, despite the necessity to spin faster, many liquid-state NMR experiments with ^1^H detections for 3D assignment of proteins have been successfully implemented (Linser et al., 2008; Barbet-Massin et al., 2014; Penzel et al., 2015). Depending on the nature of protein and availability of spinning frequencies various combinations of assignment, distance restraints and experiments bearing dynamical information can be implemented with ^1^H detection (Schanda et al., 2010; Agarwal et al., 2014; Linser et al., 2014; Fricke et al., 2017; Lakomek et al., 2017; Vasa et al., 2018). Figure 6 illustrates how recent developments in ^1^H-detected SSNMR led to the determination of the atomic resolution structure of HELLF amyloid fibrils (Daskalov et al., 2021). A set of 211 distance restraints obtained from ^1^H-detected SSNMR experiments was used to derive a 0.73 Å backbone structure.

### Dynamic Nuclear Polarization for the Enhancement of NMR Signal

The relatively low signal-to-noise ratio (SNR) in NMR spectra remains an important issue for NMR spectroscopy. One option to overcome this limitation is to employ dynamic nuclear polarization (DNP) at low (around 100 K) temperatures. In DNP, microwave irradiation techniques are used to transfer magnetization from electrons to nuclei in order to enhance the overall SNR. In order to apply DNP methods the samples have to be doped with radicals containing unpaired electrons. Practically, enhancement factors of ∼20–30 can be achieved routinely on commercial DNP spectrometers at high magnetic fields (Mandala and Hong, 2019). With DNP methods, specific distance restraints, conformational and dynamical information for different amyloid fibrils have been obtained (Debelouchina et al., 2010; Bayro et al., 2011; Potapov et al., 2015; Frederick et al., 2017). The main drawback of DNP-based SSNMR techniques for protein studies is related to the use of cryogenic temperatures, causing an inhomogeneous line broadening due to the presence of numerous protein conformations at low temperature. As a consequence, the spectral resolution in 1D and 2D experiments is often compromised. Application of spectroscopic techniques with higher dimensionality, combined with ^1^H-detection under DNP conditions, will certainly dramatically increase the capability of DNP-based SSNMR methods in the future.

### Electron Paramagnetic Resonance Spectroscopy as an Emerging and Complementary Tool to Investigate Amyloid Fibril Conformation

Using the same spin physics as NMR, electron paramagnetic resonance spectroscopy (EPR) measures electron spin properties to obtain information on the dynamics and conformation of electron spin labels (SL). When SL are tethered to specific residues, most often through site-directed mutagenesis, one can obtain information on protein structure and dynamics. EPR has become increasingly popular in the last two decades to characterize amyloid aggregate structure. Continuous-wave EPR (cwEPR) was used to map out residues forming amyloid structures, by detecting restrained dynamics (Tanaka et al., 2004) and/or inter-spin interactions originating from in register β-sheets (Der-Sarkissian et al., 2003). Der-Sarkissian et al. (2003) and Chen et al. (2007) pioneered this method and established low-resolution models of amyloid aggregates for α-synuclein, Aβ peptide (Török et al., 2002), IAPP (Jayasinghe and Langen, 2004), tau (Margittai and Langen, 2004, 2006), and orb2 (Cervantes et al., 2016). Similarly, structural elements of PrP (Cobb et al., 2007) and TTR (Serag et al., 2001) were characterized following this method. EPR was also used to track structural changes along the course of aggregation (Sepkhanova et al., 2009; Pavlova et al., 2016; Fichou et al., 2019; Zurlo et al., 2019). For example, oligomers of tau, in which SL are restrained but do not show inter-spin interactions, were observed before the formation of mature amyloid aggregates (Pavlova et al., 2016; Fichou et al., 2019).

The pulsed EPR method called double electron resonance spectroscopy (DEER) has become increasingly useful in the last decade to study amyloid structure, largely due to the development of Q-band EPR spectrometers. DEER uses dipolar coupling between two SL, often tethered to the surface of the same protein, to measure the distance between these SL. Importantly, DEER can extract distance distributions, providing a direct measurement of structural heterogeneity, which is often hard to access by other methods. DEER was for instance used to characterize tau amyloid aggregates made of recombinant protein and showed that they adopt heterogeneous structures that are drastically different from brain-derived structures (Fichou et al., 2018b). Another study showed that different tau fragments adopt different conformations, explaining the so-called cross-seeding barrier where certain segments cannot be seeded by others (Siddiqua et al., 2012). The same group further showed that single-point mutations have differential effects on the conformations of tau aggregates, thereby modulating seeding properties of the mutants (Meyer et al., 2014). DEER was further employed to show that seeding amyloid aggregation using mouse brain-extracted seeds triggers a structural convergence toward multiple well-defined conformers (Fichou et al., 2018a).

While EPR provides only approximate structural models, due to the necessity of introducing SL, it has notable strengths that make it highly complementary to SSNMR. First of all, the rapidity of measurements and the capacity to measure frozen samples makes EPR suitable to probe structural features in different conditions and at different times along aggregation pathways. Thus, where SSNMR typically provides an exhaustive and static model of the amyloid, EPR is extremely useful to characterize intermediate species and obtain a mechanistic view of amyloid formation. Furthermore, EPR obtains signal from all spins, whether they are part of insoluble or soluble assemblies. It is often possible to decompose the signal in different components, making the method capable of revealing different oligomeric species (Sepkhanova et al., 2009; Pavlova et al., 2016) and measuring the different conformations present in a heterogeneous sample (Siddiqua et al., 2012). Although some systems, such as tau, have been extensively studied in parallel by both NMR and EPR, only few studies combine the two techniques (Cervantes et al., 2016). Exploiting the complementary strengths of these two methods will be a key asset in the future to understand and characterize the formation of functional and pathological amyloids.

## Conclusion

In the past two decades, methodological advances in solid-state NMR spectroscopy have led to the development of efficient biophysical techniques to extract structural and dynamics information of protein aggregates. Amyloid fibrils, oligomers and prion aggregates arising from pathological and functional processes have been investigated by SSNMR, leading to the establishment of numerous 3D structural models. Recent methods based on fast magic angle spinning and the use of DNP have considerably increased the analytical capabilities of the technique. Integration of SSNMR structural information in combination with EPR spectroscopy and cryo-electron microscopy will provide a powerful arsenal of methods for structural biologists and biochemists to characterize complex amyloid-based assemblies.

## Author Contributions

All authors listed have made a substantial, direct and intellectual contribution to the work, and approved it for publication.

## Conflict of Interest

The authors declare that the research was conducted in the absence of any commercial or financial relationships that could be construed as a potential conflict of interest.
